# Tripartite Interplay of Epithelial, Immune, and Microbiota Dysregulation in Crohn's Disease: Mechanisms and Translational Strategies

**DOI:** 10.1002/iid3.70538

**Published:** 2026-10-06

**Authors:** Qing Lai, Qing‐Yun Zhang, Wei‐Peng Yang, Chun‐Ying Luo

**Affiliations:** ^1^ Clinical Medicine Department Youjiang Medical University for Nationalities Baise Guangxi China; ^2^ Department of Pathology Affiliated Hospital of Youjiang Medical University for Nationalities Baise Guangxi China; ^3^ Department of Science Research The Fifth People's Hospital of Nanning Nanning Guangxi China

**Keywords:** Crohn's disease, epithelial barrier dysfunction, gut microbial dysbiosis, immune dysregulation, precision therapy

## Abstract

**Background:**

Crohn's disease (CD) is a multifactorial inflammatory bowel disease (IBD) characterized by impaired epithelial barrier integrity, excessive immune hyperactivation, and gut microbiota perturbations. Cumulative evidence supports the role of the intestinal epithelial barrier, immune system and gut microbiota in the progression of CD.

**Objective:**

This review constructs a mechanistic framework of the epithelial‐immune‐microbiota tripartite network in CD with a focus on positive‑feedback circuits sustaining persistent intestinal inflammation.

**Key Findings:**

Interrelated pathological loops involving barrier injury, immune activation and microbial dysbiosis perpetuate chronic intestinal inflammation and provide a rationale for mechanism‑guided combinatorial therapeutic interventions.

**Conclusions:**

This framework illuminates key pathogenic crosstalk that may be targeted to optimize future treatment regimens for CD.

**Abbreviations**
AIEC: Adherent‐Invasive *Escherichia Coli*
ARE: AU‐Rich Element
CD: Crohn's disease
GPCR: G protein‐coupled receptor
HDAC: histone deacetylase
IBD: inflammatory bowel disease
IBD‐AIM: IBD—Artificial Intelligence Model
IL: interleukin
JAK: Janus kinase
NF‐κB: nuclear factor‐κB
NOD2: nucleotide‐binding oligomerization domain‐containing protein 2
S1P: sphingosine‐1‐phosphate
SCFA: short‐chain fatty acid
STAT3: signal transducer and activator of transcription 3
STRIDE‐II: selecting therapeutic targets in inflammatory bowel disease‐II
TGF‐β: transforming growth factor‐beta
Th17: T helper 17 cell
TLR: toll‐like receptor
TNF‐α: tumor necrosis factor‐alpha
Treg: regulatory T cell
UC: ulcerative colitis

## 1 Introduction

Crohn's disease (CD) is a complex inflammatory disorder driven by the interaction of the intestinal epithelial barrier, the immune system and the gut microbiota. Increased intestinal permeability and disruption of the epithelial barrier, as evidenced by defects in adhesion proteins such as claudins and occludins, may constitute an initiating event in disease pathogenesis. These alterations facilitate luminal antigen translocation and sustain inflammatory responses [1, 2]. Immune dysregulation in CD involves expansion of Th17 cells, IL‐17‐mediated tissue injury, and an imbalance in cytokine production, which further exacerbates mucosal injury [3]. Intestinal dysbiosis is characterized by reduced microbial diversity, expansion of potential pathobionts such as adherent‑invasive *Escherichia coli* (AIEC), and depletion of short‑chain fatty acid (SCFA)‑producing taxa, which further fuels immune activation and barrier impairment [4, 5].

Through reciprocal feedback loops, barrier impairment enables microbial and antigen translocation, triggering aberrant immune activation, which in turn compromises epithelial repair and microbial homeostasis. Several therapeutic interventions seek to modulate this interconnected pathological network, including IL‐23 inhibitors (e.g., risankizumab) to suppress Th17 cell activation, SCFA supplementation to support epithelial barrier function, and microbiota‐directed agents (e.g., probiotics) to restore microbial homeostasis. These approaches represent a transition towards mechanism‐informed therapeutic strategies [4, 6]. Emerging multi‐omics platforms, including metagenomics and serum proteomics, may further improve patient stratification, in keeping with the 2023 recommendations of the International Organization for the Study of IBD, which emphasize early intervention in high‐risk CD phenotypes [6]. Future studies should determine whether therapeutic strategies that address more than one pathogenic compartment can improve durable disease control in selected patients with CD.

Future therapeutic approaches should therefore target all three pathological compartments to improve clinical outcomes in patients with CD.

Although crosstalk among these three axes is well‑recognized, few existing studies provide mechanistic integration of their dynamic inter‑axis crosstalk. This kaleidoscopic picture masks the self‐reinforcing circuit inducing chronic inflammation. Therefore, this review summarizes the architecture of these pathogenic circuits and discusses how this framework may inform the development of multi‐target therapeutic strategies.

Rather than proposing a novel pathogenic axis, this framework highlights the dynamic coupling and stage‑dependent contributions of well‑established components and provides an integrated perspective on how pathways driving epithelial barrier impairment, immune activation, and microbial dysbiosis contribute to CD progression.

It provides a testable “tripartite feedback‐loop” framework in which epithelial barrier injury, immune activation, and microbial dysbiosis are conceptualized as interconnected nodes within a self‑reinforcing network that may exert distinct contributions across patients and at different disease stages. This framework should provide a basis for exploring whether patients with CD can be stratified into barrier‑dominant, immune‑dominant, microbiota‑dominant, or mixed biological phenotypes, an idea that could be evaluated in longitudinal clinical cohorts. Instead of a rigid, universal sequence of therapeutic choices, this framework suggests that therapeutic approaches may require prioritization according to core pathogenic features alongside serial assessments of mucosal healing, inflammatory biomarkers, and microbiota‑related indicators.

## 2 Materials and Methods

As a narrative review, this article synthesizes available evidence concerning the interplay among epithelial barrier dysfunction, immune dysregulation, and gut microbiota dysbiosis in CD. We searched PubMed and ClinicalTrials.gov for relevant literature, prioritizing mechanistic studies, clinical trials, and high‐quality reviews published from 2009 to 2025. Our synthesis focuses on three core pathogenic axes: the epithelial axis, the immune axis, and the microbial axis, with particular emphasis on their positive‑feedback circuits and therapeutic relevance.

## 3 Mechanisms of Tripartite Dysregulation

### 3.1 Epithelial Cell Dysfunction

Epithelia represent an interface between the host and the luminal environment. Dysregulation of epithelial barriers contributes to multiple pathological processes in IBD, including CD and ulcerative colitis (UC). In this section, we summarize the structure and function of the epithelial barrier, pathways that regulate the epithelial activity and epithelial cell‐crosstalk with immune cells.

#### 3.1.1 Structure and Functional Dysregulation of the Epithelial Barrier

The intestinal epithelium forms a selective barrier defined by tight intercellular junctions (e.g., claudins and occludins) as well as secreted mucosal defenses including mucins and antimicrobial peptides. Collectively, these components confer intestinal protection against pathogen invasion while permitting nutrient uptake [7, 8]. In IBD, tight‑junction disruption compromises epithelial barrier function, resulting in paracellular leakage (termed “leaky gut”), impaired mucus synthesis, microbial translocation, and aberrant immune responses [8]. Epithelial cell dysfunction can be exacerbated by pro‐inflammatory cytokines, including tumor necrosis factor‐alpha (TNF‐α) and IL‐6. A decrease in Wnt/β‐catenin signaling may also impair mucus synthesis, driving intestinal inflammation and tissue damage [9].

#### 3.1.2 Signaling Pathways in Epithelial Cell Function

Epithelial cells constantly sense and respond to environmental signals that govern their proliferation, differentiation, and stress‑response programs. The Wnt/β‐catenin, Notch and PI3K/Akt pathways are important regulators of epithelial homeostasis and injury repair [10]. For instance, Wnt signaling is essential for intestinal epithelial proliferation and differentiation, whereas Notch signaling mediates cell‑fate decisions during development and tissue homeostasis [10].

Disruption of intestinal epithelial signaling pathways is linked to aberrant epithelial‑cell behaviour in IBD. For example, activation of NF‐κB signaling during inflammatory interactions can remodel cytokine production and downregulate tight‑junction expression, thereby exacerbating epithelial‑barrier defects [8, 11]. In addition, activation of intracellular inflammatory pathways in epithelial cells may protect against inflammatory injury, underscoring the contribution of these signaling systems to epithelial‑function homeostasis [10]. Accordingly, modulation of these pathways may provide insights into strategies for restoring mucus‐layer activity and tissue regeneration, thereby supporting IBD treatment (Figure 1).

**Figure 1:**
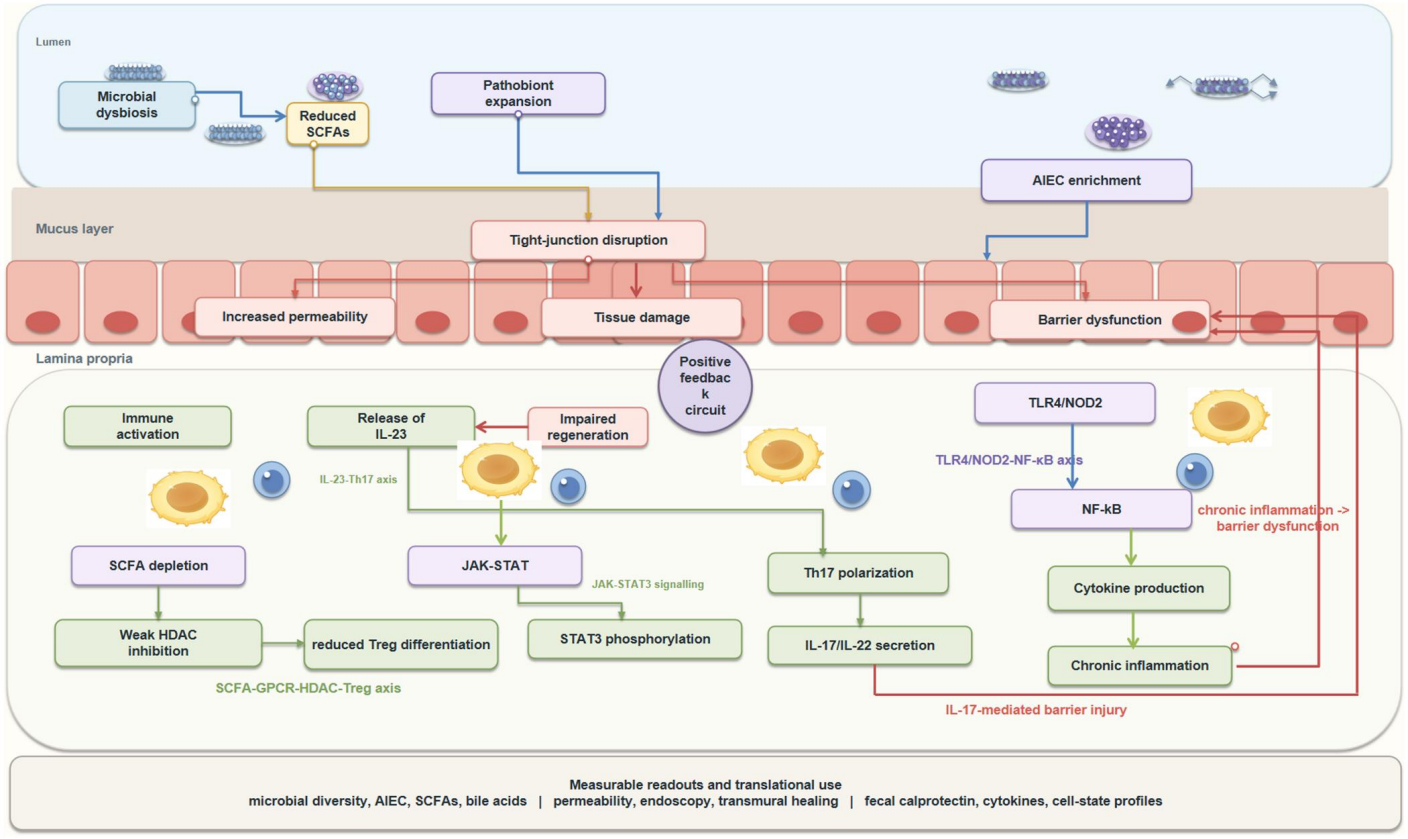
Triple inflammatory pathway involving epithelial barrier dysfunction, microbial dysbiosis and immune activation in CD. Reduced short‐chain fatty acid (SCFA) levels, expansion of pathobionts and enrichment of adherent‐invasive *Escherichia coli* (AIEC) may disrupt epithelial barrier integrity and interfere with immune regulation. Barrier disruption increases intestinal permeability and may activate TLR4/NOD2‐NF‐κB, JAK‐STAT3, and IL‐23‐Th17 signaling pathways, thereby promoting cytokine production, IL‐17‐mediated epithelial injury and chronic inflammation. These processes may further impair epithelial regeneration and exacerbate microbial dysbiosis, forming a positive feedback loop. Key translational indicators include microbial composition, SCFA and bile acid profiles, intestinal permeability, endoscopic and transmural healing, fecal calprotectin, cytokine profiles, and immune‐cell status. AIEC, adherent‐invasive *Escherichia coli*; GPCR, G protein‐coupled receptor; HDAC, histone deacetylase; IL, interleukin; NF‐κB, nuclear factor‐κB; NOD2, nucleotide‐binding oligomerization domain‐containing protein 2; SCFA, short‐chain fatty acid; STAT3, signal transducer and activator of transcription 3; Th17, T helper 17 cell; TLR4, toll‐like receptor 4; Treg, regulatory T cell. Figure 1 was created with Figdraw 2.0 by the authors for this manuscript.

#### 3.1.3 Interactions Between Epithelial Cells and Immune Cells

Interactions between intestinal epithelial cells (IECs) and immune cells governs mucosal homeostasis and immune‑response programs. Beyond their role as a physical barrier, epithelial cells actively engage in immune surveillance by secreting cytokines and chemokines that recruit immune cells to inflammatory sites. For example, during inflammation, epithelial cells can produce IL‐8 to recruit neutrophils and other pro‐inflammatory mediators that promote local immune responses [12].

In IBD, disease pathogenesis may be partially driven by disrupted epithelial‑immune crosstalk. Pathobionts such as AIEC can remodel immune‑cell recruitment and activation, resulting in deficient pathogen‑clearance responses and exacerbated inflammation [12].

A more comprehensive understanding of epithelial–immune interactions may help identify strategies to modulate their reciprocal effects and restore mucosal homeostasis in patients with IBD. For example, therapies designed to reinforce epithelial‑barrier function and boost anti‑inflammatory immune responses have been reported to ameliorate IBD‑related chronic inflammation [12].

Epithelial‐cell dysfunction is widely acknowledged as a key driver of IBD progression. Deciphering the structure‑function properties of the epithelial barrier, signaling pathways governing epithelial behaviour, and epithelial‑immune‑cell crosstalk may aid the development of therapies that foster epithelial repair and improve IBD clinical outcomes.

### 3.2 Aberrant Immune Reactions

This intricate network defends the host against pathogens while sustaining tissue homeostasis. Dysregulated immune responses can occur in situations such as autoimmune disease, chronic inflammation and immunosuppression. This section addresses the abnormal immune responses that relate to autoimmunity, imbalance of pro‐inflammation and anti‐inflammation cytokines, antigenic stimulation and recruitment of activated immune cells.

#### 3.2.1 Role of Autoimmune Mechanisms

Autoimmunity underlies numerous autoimmune disorders, in which the immune system fails to discriminate between self and non‑self. In autoimmune disorders, the immune system targets host tissues, triggering inflammation and tissue injury. Autoimmune diseases represent multifactorial conditions shaped by genetic predisposition, dysregulated immune responses, and environmental triggers. For instance, systemic lupus erythematosus is a multisystem autoimmune disorder driven by loss of tolerance to nuclear antigens, systemic inflammation, and multi‑tissue injury.

T and B cells have coordinated roles in autoimmunity: activated T cells may drive inflammation, whereas B cells produce autoantibodies that contribute to tissue injury. Regulatory T cells (Tregs) are also important for maintaining immune tolerance, and their dysfunction has been linked to loss of tolerance and the initiation of autoimmune diseases [13]. Autoimmune responses may also be fueled by crosstalk between genetic predisposition and environmental exposures, including infections and xenobiotics. For example, certain autoimmune conditions are associated with distinct infectious agents, supporting the notion that molecular mimicry can trigger autoreactive immune‑cell activation. Elucidation of these mechanisms may inform the development of targeted therapeutic strategies to restore immune tolerance while mitigating tissue injury.

#### 3.2.2 Imbalance of Pro‐ and Anti‐Inflammatory Cytokines

Balance of cytokines is crucial for physiological immune responses. Disbalance between pro‑ and anti‑inflammatory cytokines can exacerbate chronic inflammation and amplify disease severity across autoimmune disorders, cardiovascular diseases, and metabolic syndrome. Pro‑inflammatory cytokines such as TNF‑α and IL‑6 are frequently upregulated under inflammatory conditions; they drive immune‑cell activation and amplify tissue injury. By contrast, anti‐inflammatory cytokines (e.g., IL‐10 and transforming growth factor [TGF]‐β) can suppress inflammation and facilitate tissue repair.

Excessive production of pro‑inflammatory cytokines such as TNF‑α and IL‑6 drives chronic inflammation, and this pathogenic role has been therapeutically validated using cytokine inhibitors [14]. Restoring inflammatory balance without damaging protective immune function against infection remains a major therapeutic challenge. Research into signaling pathways governing cytokine production and immune‑cell function is therefore indispensable.

#### 3.2.3 Mechanisms of Immune Cell Activation and Recruitment

Immune‐cell activation and recruitment are central features of host immune responses that allow for effective responses to pathogens and tissue injury. These processes are orchestrated by diverse signals, including cytokines, chemokines, and pathogen‑associated molecular patterns. Macrophages, dendritic cells, T cells, and other immune‑cell subsets are pivotal for pathogen sensing and the initiation of immune responses.

Chemokines play a particularly critical role in directing immune‑cell migration towards sites of inflammation or infection. For instance, CCL2 and CXCL10 recruit monocytes to inflammatory sites and T cells to sites of viral infection, respectively. Crosstalk between chemokines and their receptors on immune cells is essential for immune surveillance and responsiveness [15]. Dysregulated chemokine‑driven immune‑cell recruitment can compromise immune surveillance and fuel chronic inflammation.

Recent studies have highlighted the role of pathobionts in shaping immune‑cell development, recruitment, and activation. Pathobionts can establish an immune microenvironment that blunts protective host responses to pathogens. Dissecting how pathobionts modulate immune‑cell development and function may inform immunotherapeutic approaches to boost protective immune responses [16].

Overall, dysregulated immune responses involve complex and multifaceted mechanisms related to mucosal immunity, cytokine balance and immune‐cell activation and recruitment. Further studies are required to clarify how these immune alterations interact with epithelial dysfunction and microbial dysbiosis during different stages of CD.

### 3.3 Changes in Microbiota

The human microbiota, particularly the gut microbiota, represents a key modulator of host health, exerting broad‑ranging effects on diverse physiological processes including immunity, metabolism, and host behavior. Alteration of gut microbiota composition and function has been revealed to mediate multiple diseases such as IBD, obesity and metabolic syndrome [17]. This overlap is relevant to CD because metabolic comorbidities, particularly Type 2 diabetes, may influence disease course and treatment patterns. Shared pathways involving dysbiosis, epithelial barrier impairment, altered microbial metabolites and chronic low‐grade inflammation may also provide a mechanistic basis for further investigation [18]. Thus, metabolic disorders should be considered potential modifiers of the epithelial‐immune‐microbiota axis, not merely isolated comorbidities.

#### 3.3.1 Composition of Gut Microbiota

The gut microbiota constitutes a diverse microbial community forming a complex intestinal ecosystem, predominantly comprising bacteria, archaea, viruses, and fungi. Human gut‑microbiota profiles vary markedly between individuals, shaped by host genetics, diet, lifestyle, and environmental exposures. Dysbiosis, defined as imbalance of gut microorganisms, can contribute to a number of diseases, including IBD, such as CD and UC. In CD, for instance, multiple studies have documented microbial dysbiosis characterized by reduced microbial diversity, dominance of selected bacterial taxa, and concomitant depletion of core beneficial members, particularly SCFA‐producing representatives of the phylum Firmicutes, together with variable alteration of Bacteroidetes [19].

As outlined above, diet represents a key modulator of gastrointestinal microbiota composition. Fiber‐rich diets promote the expansion of beneficial genera including *Bifidobacterium* and *Lactobacillus* that ferment the fiber to SCFAs and help maintain gut barrier function and immune regulation [20]. By contrast, high intake of processed foods and rich‐food‐based sugar‐rich diets can favor the expansion of pathobionts, exacerbate inflammation, and promote disease onset [21]. Understanding how specific changes in microbiota composition relate to different diseases can inform intervention strategies for maintaining microbial balance and overall wellbeing, including dietary reform and probiotics.

#### 3.3.2 Impact of Microbial Metabolites

Microbial metabolites, particularly host‑beneficial SCFAs, constitute key mediators of microbiota‑host crosstalk. SCFAs (e.g., acetate, propionate, and butyrate) are end‐products of fermentation of dietary‐fiber by the gut microbiota, and exert anti‑inflammatory and immunomodulatory properties [22]. For example, butyrate is a primary energy source for colonic epithelial cells and sustains gut‑barrier integrity. Dysregulated SCFA production drives gut‑barrier dysfunction and contributes to IBD and other intestinal disorders [22].

SCFAs also exhibit immunomodulatory potential in CD. For example, butyrate can exert an anti‐inflammatory effect by promoting differentiation of regulatory T cells (Treg cells) and have a counter anti‐inflammatory effect by inhibiting Th17 pro‐inflammatory T helper cells in an HDACs (histone deacetylases) dependent manner [23]. SCFAs can additionally protect intestinal‑barrier function by upregulating tight‑junction expression and innate immune responses within epithelial cells [24]. These findings support the potential relevance of SCFAs for CD therapy and highlight plausible mechanisms involving immune and epithelial‑cell modulation.

Beyond SCFAs, other microbial metabolites such as bile acids, tryptophan derivatives, amino acid derivatives, can affect host metabolic functions and immunity. Bile acids are synthesized from cholesterol in the liver, and they can be modified by gut bacteria, and so regulate lipid metabolism and glucose metabolism. Tryptophan derivatives likewise shape host immunity and neurobehavioural status [25]. SCFAs, for example, the SCFA butyrate reinforces epithelial‑barrier integrity via tight‑junction upregulation [24] and suppressing Th17‑cell responses.

#### 3.3.3 Interaction Between Microbes and Host Immunity

In CD, dysbiosis disrupts the microbiota‑immune axis. Reduced SCFA availability impairs Treg‑mediated immune tolerance [22], whereas pathobionts such as adherent‑invasive *Escherichia coli* (AIEC) trigger TLR/NF‑κB signaling [26], and induce IL‐17 and TNF‐α production [27]. Although debate persists regarding the dual role of dysbiosis in disease onset and disease progression, the prevailing consensus holds that dysbiosis acts merely as a modulator rather than a primary initiating trigger.

Conversely, dysbiosis drives immune dysregulation, which in turn fuels the progression of autoimmune and chronic inflammatory disorders. For example, in patients with CD, immune responses may shift into a proinflammatory state and decrease in Treg activity [20]. Furthermore, given the the link between specific microbial taxa and improved responses to immune‑checkpoint inhibitors, microbiota‑dependent regulation of cancer immunotherapy has been extensively studied, highlighting therapeutic prospects for microbiota‑targeted interventions [28].

Collectively, existing evidence underscores the importance of understanding gut microbial composition, microbial metabolism, and host–microbiota interactions in CD. Further mechanistic studies are needed to determine how these interactions contribute to disease progression and treatment response.

### 3.4 Synthesis of Mechanistic Insights and Therapeutic Implications

Thus, the preceding sections indicate that epithelial‑barrier dysfunction, immune dysregulation, and gut microbial dysbiosis represent interrelated facets of a self‑propagating pathogenic circuit in CD, rather than discrete disease manifestations. The key mechanistic insight is that the disruption of any of these sides of the circuit—a broken epithelium, for example—may aggravate the others, including immune activation and dysbiosis, and reinforce the primary failure. This vicious‑cycle model carries critical therapeutic implications. Specifically, although single‐pathway therapies can be highly effective in selected patients, persistent activity in other pathogenic compartments may help explain incomplete or non‐durable responses in some cases. Conversely, therapeutic interventions that target multiple circuit components either concurrently or sequentially (including epithelial repair, immune recalibration, and restoration of microbial homeostasis) may help break this pathogenic circuit [6]. Subsequent sections elaborate on the specific feedback loops within this tripartite regulatory network (Section 3.5) and examine how this holistic framework can inform the development of precision‑targeted therapies (Section Key Findings, 3).

### 3.5 Interrelationships of Tripartite Dysregulation

Interactions among intestinal immune microenvironment, epithelium barrier, immune system, and microbiota are altered in IBD (especially CD and UC), and probably central in the pathogenesis of IBD. Understanding regulatory crosstalk between these components is critical for unravelling disease‑pathogenesis mechanisms and identifying viable therapeutic strategies. Disruption of the epithelial–immune–microbiota network may disturb intestinal homeostasis, giving rise to chronic inflammation, epithelial‑barrier impairment, and gut microbial dysbiosis. These components exert bidirectional influences on one another, forming positive‑feedback loops that govern inflammatory‑response magnitude and disease progression (Table 1) [29].

**Table 1:** Three key positive feedback loops in Crohn's disease.

Feedback loop	Core mechanism	Experimental models	Therapeutic targets	Key references
IL‐23/Th17 Axis	IL‐23 drives Th17 polarization, leading to IL‐17 secretion, epithelial barrier disruption, microbial translocation, and sustained IL‐23 release.	TNFΔARE mouse model (Th17 expansion); Single‐cell sequencing in CD patients (Th17/Treg imbalance).	IL‐23 inhibitors (e.g., risankizumab); JAK/STAT inhibitors (e.g., upadacitinib, filgotinib).	[6, 30]
SCFA Depletion Cycle	Reduced SCFA levels impair Treg differentiation, promoting Th17 dominance, inflammation, and further depletion of SCFA‐producing microbiota.	Germ‐free mice supplemented with SCFAs; Fecal metabolomics in CD patients (low butyrate).	SCFA supplementation; High‐fiber diets; Probiotics targeting SCFA‐producing taxa.	[22, 23, 24]
TNF‐α‐Driven Pathway	TNF‑α disrupts epithelial tight junctions, enabling pathobiont translocation, TLR4/NF‑κB activation, and amplified TNF‑α production.	TNFΔARE mice; Patient‐derived intestinal organoids; Postoperative microbiota‐metabolome studies.	Anti‐TNF‐α biologics (e.g., infliximab); Gut‐selective S1P modulators; Microbiota‐targeted diets.	[6, 20, 30, 31]

*Note:* 1. IL‐23/Th17 Axis: IL‐23‐mediated Th17 polarization leads to ongoing epithelial damage and dysbiosis, creating a self‐perpetuating inflammatory cycle. 2. SCFA Depletion Cycle: A lack of SCFAs disrupts immune tolerance and the integrity of barriers, worsening dysbiosis and inflammation. 3. TNF‐α‐Driven Pathway: TNF‐α‐induced barrier dysfunction allows for the translocation of pathobionts, which heightens innate immune activation.

Abbreviations: ARE, AU‐Rich Element; CD, Crohn's disease; IL‐23, interleukin‐23; JAK‐STAT, Janus kinase‐signal transducer and activator of transcription; NF‐κB, nuclear factor‐κB; SCFA, short‐chain fatty acid; S1P, Sphingosine‐1‐phosphate; Th17, T helper 17 cells; TLR, toll‐like receptor; TNF, tumor necrosis factor; Treg, regulatory T cell.

#### 3.5.1 Feedback Mechanisms Between Epithelium and Immunity

IECs sustain mucosal homeostasis by segregating luminal microbiota away from the immune system via tight junctions, antimicrobial peptides, and mucus secretion. In IBD, altered pattern‐recognition receptor signaling, including toll‐like receptor (TLR) activation, may impair this barrier and promote excessive production of pro‐inflammatory cytokines and recruitment of immune cells to inflammatory sites [29]. In addition, diminished secretion of protective mediators such as secretory IgA and defensins, coupled with elevated mucosal permeability, enables bacterial‑antigen translocation, which further amplifies immune activation and aggravates mucosal inflammation [28].

The microbiota also shapes epithelial–immune interactions. Dysbiosis can trigger aberrant immune activation and compromise protective barrier function [29]. Thus, crosstalk between the epithelium and the immune system is essential for maintaining intestinal homeostasis, whereas its disruption can initiate or drive IBD pathogenesis.

#### 3.5.2 Regulatory Roles of Microbiota

The gut microbiota acts as a key regulator of immune responses and intestinal homeostasis. Available data demonstrate that beneficial bacterial taxa are depleted while potential pathobionts are enriched within the gut microbiota of patients with CD [32].

The microbiota can modulate the immune system through the production of SCFAs generated by dietary‐fiber fermentation. SCFAs, most notably butyrate, exert anti‑inflammatory properties and are critical for sustaining epithelial‑barrier integrity. These metabolites can further modulate immune‑cell development and function and reinforce Treg‑dependent immune tolerance [33].

Dysbiosis can fuel a pro‑inflammatory positive‑feedback loop across the microbiota‑immune interface (Figure 2). For example, certain pathobionts, such as AIEC, can induce pro‐inflammatory cytokine production and perturb the epithelial barrier, generating a vicious cycle linking inflammation and microbial dysbiosis [29]. Furthermore, depletion of beneficial gut commensals compromises SCFA synthesis, which further worsens impairment of epithelial‑barrier integrity and immune homeostasis. Collectively, these interactions among the microbiota, immune system, and epithelium highlight the importance of a balanced gut microbiota to maintain intestinal health.

**Figure 2:**
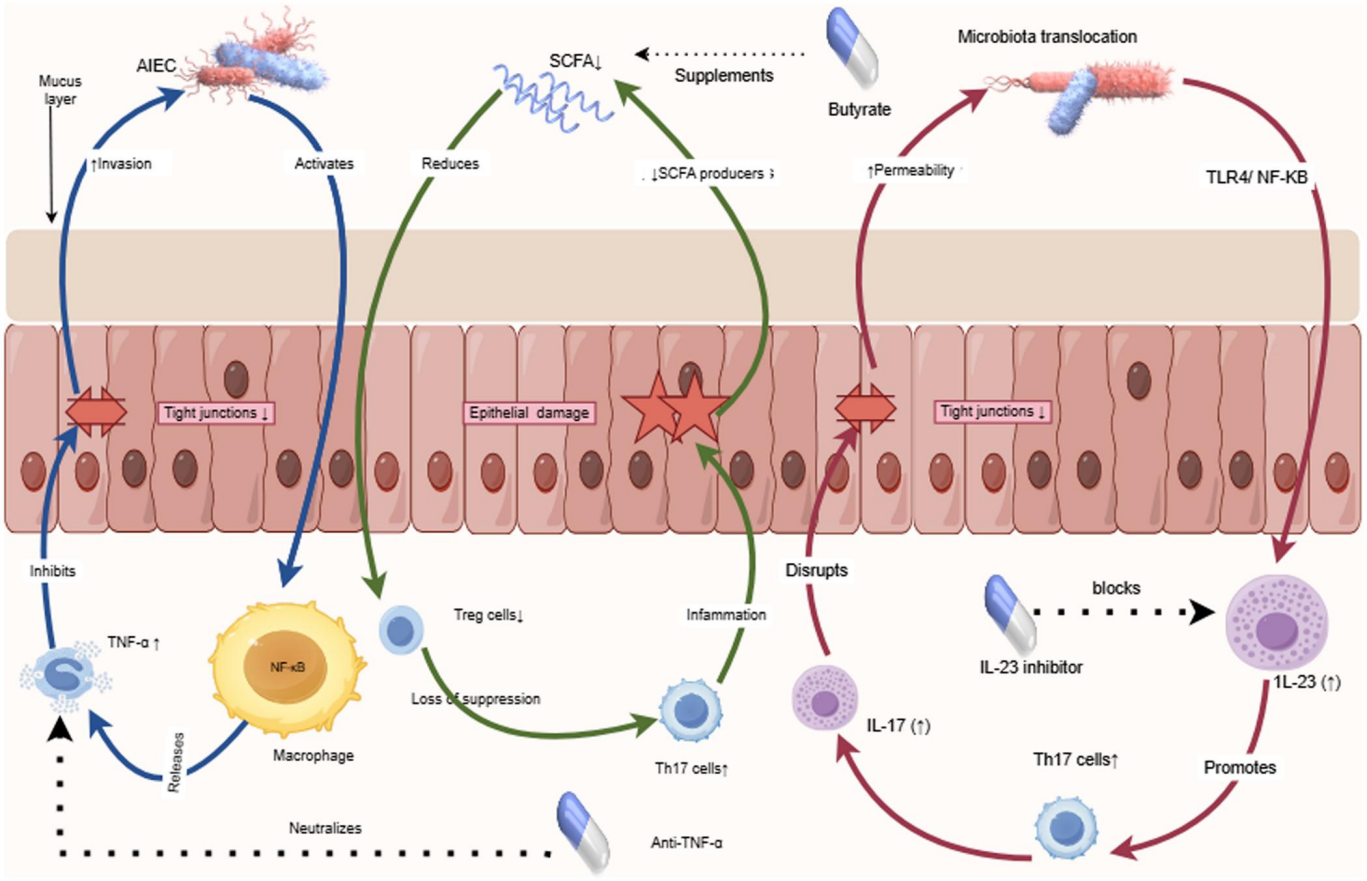
Tripartite pathogenic network and targeted therapies in CD. Solid lines denote direct promotion or inhibition, whereas dotted lines represent therapeutic interventions. The core pathogenic circuits comprise the IL‑23/Th17 axis, TNF‑α feedback loop, and SCFA depletion cycle. In the IL‐23/Th17 axis (red arrows), increased IL‐23 expression promotes Th17‐cell differentiation and disrupts tight junctions, thereby permitting microbial translocation that may reactivate IL‐23 signaling. In the TNF‑α feedback loop (blue arrows), TNF‑α down‑regulates junctional protein expression, facilitating AIEC invasion, macrophage NF‑κB activation, and subsequent TNF‑α production. In the SCFA depletion cycle (green arrows), diminished butyrate levels can impair Treg differentiation and exacerbate epithelial injury. Therapeutic interventions include IL‐23 blockade to inhibit Th17 polarization, anti‐TNF‐α therapy to neutralize TNF‐α and butyrate supplementation to restore Treg homeostasis. AIEC, adherent‐invasive *Escherichia coli*; HDAC, histone deacetylase; NF‐κB, nuclear factor‐κB; SCFA, short‐chain fatty acid; TLR, toll‐like receptor; Treg, regulatory T cell. Figure 2 was created with Figdraw 2.0 by the authors for this manuscript.

#### 3.5.3 Network Effects of Tripartite Dysregulation

Disrupted homeostasis among the intestinal epithelium, immune system, and microbiota gives rise to an interconnected regulatory network that governs disease progression and resolution in IBD. In this network, perturbation of one component propagates disturbances across the remaining compartments, perpetuating inflammation and tissue injury. For example, intestinal epithelial barrier changes may also increase the intestinal epithelium barrier permeability, thereby allowing microbial antigens to enter the lamina propria and trigger the immune system. Subsequent immune activation further aggravates epithelial injury, establishing a positive‑feedback loop between inflammation and dysbiosis [8].

Environmental factors, including diet and stress, may also affect network effects of tripartite perturbation by modulating microbiota composition and immune responses. For example, high‑fat diets expand pro‑inflammatory bacterial taxa and deplete beneficial commensals, driving compositional shifts within the gut microbiota [17]. Psychological stress can likewise reduce mucosal immune competence and promote dysbiosis, thereby exacerbating inflammation in patients with IBD [34].

Integrating these three factors into strategies for restoring intestinal balance, such as probiotics, dietary modification and immune regulation, holds therapeutic promise for IBD management [25]. Elucidating the network consequences of tripartite imbalance can also facilitate the design of more holistic therapeutic approaches for IBD.

It is also important to research the network effects arising from tripartite imbalance is also critical for designing more comprehensive therapeutic interventions for IBD.

### 3.6 Research Progress of Novel Mechanisms

#### 3.6.1 Findings From Animal Models

Animal models serve as valuable tools for dissecting SCFA‑related mechanisms. For example, mouse models of telomere dysfunction, for example, activation of ATM/YAP1/pro‐IL‐18 pathway, support the potential role of DNA‐damage signaling in the pathogenesis of CD‐associated inflammation [35]. The TNFΔARE mouse model, which recapitulates key features of human CD including microbial dysbiosis and immune hyperactivation, provides a platform to investigate antigen‑driven disease relapse [30]. Likewise, rodent experiments demonstrate that AIEC colonization potentiates intestinal inflammation and reproduces key clinical manifestations of CD [36]. Other novel models such as the perianal fistulizing CD model will open new capabilities in preclinical models [37]. Together, these systems foster synergies between mechanistic discoveries and translational therapeutic development.

#### 3.6.2 Application of Modern Omics Technologies

Recent progress in omics technologies has have transformed research approaches to CD, enabling global‑scale profiling of genetic, epigenetic, transcriptomic, proteomic, and metabolomic alterations. The identification of reliable biomarkers may facilitate prediction of disease activity and treatment response, supporting the development of personalized medicine for CD. For example, multi‐component studies can also be applied to detecting mutual interactions between the gut microbiota, the host genetics and immunity in patients with CD. In a recent investigation with shotgun metagenomics and metabolomics to analyze the composition of the microbiota in patients with CD, microbial diversity differed between diseased patients and healthy subjects. Multiple prior reports have also linked specific bacterial‑community configurations and metabolite profiles to surgical outcomes in CD [31]. These findings advance our understanding of how microbiome diversity shapes CD pathogenesis and inform the design of microbiota‑targeted therapeutic interventions.

Proteomic studies may also allow the identification of differentially expressed proteins in immune and epithelial cells isolated from patients with CD, some of which may represent actionable therapeutic targets [38]. Multi‑omics integration facilitates comprehensive disease‑signature characterization and reveals core pathways driving intestinal inflammation and fibrosis. For example, butyrate (SCFA from the gut microbiota) has been investigated for the strength it imparts to epithelial barrier and immune regulation in CD using multi‐layer omics analyses [39]. Additionally, machine‑learning workflows applied to large‑scale omics datasets hold promise for discovering novel CD‑associated biomarkers and therapeutic targets. These advances, collectively, can not only bridge existing knowledge gaps in CD pathogenesis, but also support the development of better‑informed therapies aimed at improving clinical outcomes.

#### 3.6.3 Latest Clinical Research Results

Substantial progress has been achieved for CD, especially regarding disease pathogenesis, therapeutic strategies, and the role of lifestyle in disease management. More attention has been placed on personalized therapeutic approaches that account for inter‑patient variability, including genetic susceptibility and disease phenotype.

A promising research domain centers on novel biologic and small‑molecule agents, including Janus kinase (JAK) inhibitors. Corticosteroid‐refractory CD may provide the most relevant setting to evaluate such therapies. Upadacitinib, an oral selective JAK1 inhibitor, demonstrates efficacy for both induction and maintenance therapy in moderate‑to‑severe CD [40]. IL‐23 inhibitors, such as risankizumab, will be able to block Th17‐mediated inflammation. In ADVANCE/CD‐101, risankizumab induced greater endoscopic response than placebo at week 52 in patients with moderate‐to‐severe CD and particularly in those with anti‐TNF failure (RR: 3.40, 95% CI: 2.57–4.50; *p* < 0.001) [6]. Phase 3 induction trials further confirm risankizumab efficacy for moderate‑to‑severe CD, validating the therapeutic potential of IL‑23 inhibition [41]. For perianal CD, darvadstrocel‑based adipose‑derived stromal‑cell therapy achieved 56% fistula resolution at 24 weeks in the randomized ADMIRE‑CD II trial and has obtained marketing authorization within the EU and Japan [42].

Diet management is also an active area of investigation in CD research. The CD Exclusion Diet, which eliminates specific food components, has demonstrated efficacy for inducing and sustaining clinical remission in randomized trials among patients with mild‑to‑moderate CD across 12–24‑week follow‑up periods [43]. This dietary strategy can mitigate intestinal inflammation and sustain remission, highlighting the potential benefits of dietary modification combined with pharmacological therapy.

Advanced imaging techniques, including intestinal ultrasonography, have also been developed as non‐invasive tools for diagnosis and also for the prediction of disease activity and postoperative recurrence. Phase 3 findings for upadacitinib further support the use of oral small‑molecule therapies in selected patients with moderate‑to‑severe CD [40]. These advances facilitate a more proactive, personalized clinical‑care paradigm with encouraging prognostic potential.

Overall, clinical research in CD is still going forward, and greater understanding of the disease has led to the emergence of new treatment strategies. Omics‑based tools, personalised‑medicine workflows, and dietary interventions generate new opportunities for managing this multifactorial disorder. These clinical studies show progress not only in understanding and treating CD but also in the ongoing changes to the treatment strategies. Table 2 exemplifies the evolutionary shift from conventional management toward modern strategies built upon the proposed tripartite pathogenic framework for CD, highlighting key advances across all therapeutic domains.

**Table 2:** Evolution of therapeutic strategies in CD.

Therapeutic aspects	Traditional approaches	Emerging strategies informed by the tripartite framework	Key advancements	References
Primary focus	Single‐target immune suppression (e.g., anti‐TNF‐α)	Integrated targeting of immune activation, epithelial barrier injury and microbial dysbiosis	Mechanism‐informed approaches may help identify patients who could benefit from combined immune‐ and microbiota‐oriented strategies.	[6, 39]
Mechanistic basis	Broad immune inhibition for symptomatic control	Pathogenesis‐driven repair targeting barrier, immune, and microbial compartments	SCFAs promote Treg differentiation and epithelial barrier integrity	[23]
Target precision	Systemic immunosuppression with off‐target effects	Targeted cytokine pathway inhibition (e.g., IL‐23 blockade)	IL‐23 pathway targeting with risankizumab has shown efficacy in moderate‐to‐severe CD	[41]
Personalization	“One‐size‐fits‐all” dosing	Multi‐omics stratification (microbiota, immune, epithelial markers)	AI‐ and multi‐omics‐based models may support treatment‐response prediction, but require prospective validation before routine clinical use.	[44, 45]
Combination therapies	Empirical sequential administration of immunosuppressive agents	Rational combination regimens (biologics plus mucosal‑repair or microbiota‑modulating interventions)	Rational combinations of biologics with barrier‑ or microbiota‑targeted interventions remain conceptually promising yet require controlled clinical validation	[6, 38]
Validation status	Endorsed by clinical guidelines yet limited in achieving mucosal healing	Phase III trials for “transmural healing” as primary endpoint	STRIDE‐II supports treat‐to‐target care, with endoscopic healing as a key target and transmural assessment considered in selected settings	[6, 46]

Abbreviations: CD, Crohn's disease; JAK, Janus kinase; STRIDE‐II, selecting therapeutic targets in inflammatory bowel disease‐II; TNF, tumor necrosis factor.

Notwithstanding improvements in systemic therapeutic options, CD remains challenging to manage owing to its complex aetiology, progressive disease trajectory, and heterogeneous treatment‑response profiles. The longstanding challenges are diagnostic delay, incomplete mucosal healing and loss of response to biologic therapy. Future initiatives will seek to address early intervention, treat‐to‐target approach, novel therapies (e.g., interleukin 23 and Janus kinase inhibitors), biomarker‑guided personalised care, and microbiota‑modulating interventions, with the overarching goal of enhancing long‑term clinical outcomes and quality of life [19].

## 4 From Pathogenic Crosstalk to Precision Interventions

To operationalize this tripartite model, we conceptualized CD progression as a dynamic feedback loop with three measurable components: epithelial barrier function, immune inflammatory activity and microbial disturbance/metabolic disturbance. This model is amenable to testing because each component can be paired with observable and measurable outcomes (intestinal permeability markers, endoscopic or transmural healing, cytokines, fecal calprotectin, microbial diversity, pathobiont enrichment and microbial metabolites, for example, SCFAs [6, 7, 31]. Longitudinal profiling of these components can reveal whether disease activity is driven by sustained immune activation, persistent barrier impairment, microbial‑metabolic perturbations, or crosstalk among these factors. In patients with obesity, metabolic syndrome or Type 2 diabetes, this view also helps us evaluate whether peripheral metabolic inflammation directly modulates mucosal immune activation, as well as microbial effects on epithelial‑barrier integrity.

### 4.1 Summary of Key Findings

CD is a complex, heterogeneous inflammatory disorder defined by epithelial‑barrier dysfunction, immune hyperactivation, and microbial dysbiosis. This mechanistic review integrates these three core components into a feedback‑loop model that explains how these interrelated perturbations act in concert to perpetuate CD pathogenesis. The translational relevance of this model lies in its implication that mechanistic heterogeneity can support future‑treatment stratification. Patients with high inflammatory activity would likely be the ones to benefit from immune‐targeted interventions (including IL‐23, JAK or other cytokine‐directed) therapies [19, 40, 41]. By contrast, patients with persistent barrier dysfunction may be the ones that would likely benefit from therapies that promote repair of mucosal and transmural pathologies [7], while individuals with marked microbial instability represent suitable candidates for dietary‑, microbiota‑, or broader metabolic‑modulating strategies [20, 36]. Thus, the tripartite model should be regarded as a hypothesis‑generating construct requiring prospective validation, rather than a formal clinical‑practice guideline.

Intestinal surface epithelium forms a physical barrier against microbial invasion and constitutes a central pillar of mucosal homeostasis. In CD, the disruption of epithelium can increase the intestinal permeability and lead to a microenvironment that facilitates antigen penetration, resulting in abnormal immune activation as well as the microbiota dysbiosis. This triad pathogenesis (i.e., epithelial dysfunction, immune activation, and microbiota dysbiosis) contributes to chronic inflammation in CD [6]. Additional studies should characterize dynamic interconnections among these compartments, and how microbial shifts modulate epithelial status and immune responsiveness. Such mechanistic links may enable genetic‑ and environmental‑factor‑based risk stratification to identify high‑risk patients and inform preventive measures. Therapeutic interventions aiming to counteract tripartite dysregulation, including probiotic supplementation to restore commensal balance and dietary strategies to reinforce epithelial‑barrier integrity, hold promise for comprehensive CD management.

Personalized treatment guided by genetic susceptibility and microbial profile will also transform our treatment of CD. A major ongoing research challenge is to reconcile competing mechanistic interpretations and synthesize existing experimental evidence. Some studies highlight the role of the microbes in avoiding relapse, whereas other studies focus on the epithelial barrier defect or an immune‐mediated injury. Future research should integrate these disparate observations into a more holistic disease model for CD and foster cross‑disciplinary dialogue among microbiota, immunology, and epithelial‑biology researchers.

In summary, the tripartite dysregulation model for CD outlined above provides a valuable conceptual framework for interpreting CD pathogenesis. Improved characterization of these complex reciprocal interactions will facilitate the discovery of novel therapeutic targets and, ultimately, enhance prognosis and quality of life among patients with CD.

### 4.2 Experimental Models and the Limits of Translation

Experimental models have been invaluable for dissecting epithelial, immune, and microbial mechanisms underlying CD; nevertheless, these represent reductionist systems that cannot fully recapitulate human disease. Models such as TNFΔARE mice, mice colonized with AIEC, rodent models of chemically induced injury all capture parts of CD (such as cytokine‐evoked inflammation, pathobiont growth or barrier damage), but none of these models captures all of the polygenic susceptibility, environmental exposures, microbial diversity, transmural inflammation, skip lesions and relapsing‐remitting progression that are exhibited by patients with CD [30, 36, 37]. This consideration is critical, since a pathway that dominates in simplified preclinical systems may constitute only one component within the broader pathogenic network in human CD. Experimental findings should therefore be regarded as hypothesis‑generating and require validation in complementary translational platforms, including patient‑derived organoids, microbiota‑humanized animal models, multi‑omics cohorts, and longitudinal clinical biospecimens.

### 4.3 Controversies and Future Directions

Despite major advances, key controversies persist, most notably whether gut dysbiosis represents a primary or secondary event in CD pathogenesis. This ongoing debate draws support from disparate lines of experimental evidence. The “causative‑role” hypothesis is underpinned by preclinical data demonstrating that pathobionts such as AIEC can disrupt intestinal‑barrier integrity and trigger pro‑inflammatory Th17‑cell responses. They might be also relatively overrepresented in some patients with CD, which occurs before or in parallel to onset of disease [36]. However, these data are mainly of correlative nature, model dependent and may be limited in generalization to human disease. On the contrary, the “reactive” view stresses that this intestinal inflammatory environment, which comprises a shift in oxygen tension and of antimicrobial peptides, can strongly alter the microbial composition, favoring facultative over obligate anaerobes [47]. Effective anti‑inflammatory therapy can partially reverse dysbiosis. While this observation supports a reactive component of dysbiosis, it does not rule out contributions of dysbiosis toward post‑treatment relapse. A more integrative picture holds that dysbiosis serves as a disease‐perpetuating amplifier in the genetic‐immune‐epithelial setting, whereby the initial dysbiosis (caused by environmental triggers) and inflammation find a feed‐forward loop [29]. Overall, an integrative picture fits the data best. Dysbiosis may function as a primary pathogenic driver in young‑onset or treatment‑naïve CD, whereas it acts predominantly as a secondary phenomenon in long‑standing disease, once inflammation has established a permissive microbial niche.

Therapeutic implications diverge accordingly. If gut dysbiosis indeed exerts a primary causal role in selected patients, this warrants further evaluation of targeted microbiota manipulation—including pathogen‑specific antimicrobials and tailored probiotics—as an adjunctive or mechanism‑informed therapeutic strategy [48]. Whereas, if dysbiosis is in large part secondary to an established inflammatory process, immunomodulation remains central to primary therapy, while microbiota‑targeted interventions may serve as an add‑on to restore mucosal homeostasis. In real‑world clinical practice, this distinction would inform patient stratification. Future combination therapeutics should therefore take into account both aspects and be adapted for particular disease endotypes [6].

Future research will need precision medicine and early intervention as priorities. Integration of metagenomics and proteomics within multi‑omics platforms will facilitate patient stratification and enable personalized therapeutic strategies [44]. AI‑driven dynamic treatment models can also support clinical decision‑making for CD management. Machine‐learning algorithms can be used by combining multi‐omics data (including genomic and transcriptome data) and clinical characteristics to predict the response to treatment and to adjust treatment over time. For instance, a machine‐learning model has been developed to predict endoscopic response to ustekinumab based on genomic and transcriptomic data to help personalized treatment selection [45]. Further work is required to refine these models and enhance their predictive performance and translational potential. Challenges however include the standardization and costs of microbiota‐directed interventions. These barriers may be addressed through international collaborative efforts such as the IBD Character Initiative [49].

### 4.4 Translational Implications of the Tripartite Framework

STRIDE‐II recommendations emphasize treat‐to‐target monitoring, including endoscopic healing and, in selected contexts, transmural assessment using intestinal ultrasound or MRI [46]. AI‑driven platforms such as IBD‑AIM increasingly leverage multi‑omic datasets to forecast therapeutic responses and facilitate adaptive therapeutic adjustments as required [44, 45]. Artificial intelligence‐assisted endoscopy systems, including EndoBrain and CAD‐EYE, integrate endoscopic imaging‑derived radiomics with intestinal mucosal transcriptomic data and constitute advanced artificial‑intelligence applications for endoscopic IBD assessment [44]. These systems can enhance the detection of dysplastic and inflammatory lesions in patients with IBD while lowering inter‑observer variability. At present, such technologies are primarily deployed for long‑term monitoring of patients with IBD and for supporting clinical‑trial assessments, and have been implemented at leading medical centers across Europe and North America [50]. Nonetheless, a substantial translational gap remains. Numerous findings related to epithelial, immune and microbiota factors are not yet sufficiently robust to guide treatment selection for individual patients. This limitation arises partly because most biomarkers capture only one dimension of disease biology, whereas cross‐sectional observations cannot reliably distinguish primary disease drivers from secondary consequences. Future clinical translation will necessitate longitudinal validation, the development of integrated biomarker panels, and trials testing whether mechanism‐guided treatment improves mucosal/transmural healing, lowers relapse risk, and delivers better therapeutic outcomes than conventional clinical stratification [29, 31, 44].

## Author Contributions

Q.L. conceived the review, designed its methodological framework, conducted the literature investigation and evidence synthesis, curated information from the published literature, drafted the manuscript, and generated the figures. Q.‐Y.Z. contributed to methodology design, literature investigation, and data curation. W.‐P.Y. contributed to literature investigation and critically reviewed and revised the manuscript, with special focus on clinical relevance. C.‐Y.L. acquired funding, supervised the work, administered the project, and coordinated manuscript preparation. All authors have read and approved the final version of the manuscript for submission.

## Conflicts of Interest

The authors declare no conflicts of interest.

## Data Availability

This narrative review generates no new datasets. All information presented herein is derived from publicly available literature cited in the reference list.
